# Vascular Endothelial Growth Factor A (VEGFA) Regulates Hepatic Lipid and Glycogen Metabolism in *Schizothorax prenanti*

**DOI:** 10.3390/ijms242015171

**Published:** 2023-10-14

**Authors:** Yan Wang, Jiahui Ni, Aiyu Wang, Run Zhang, Linjie Wang

**Affiliations:** College of Animal Science and Technology, Sichuan Agricultural University, Chengdu 611130, China; 201605189@stu.sicau.edu.cn (J.N.); w1771692360@163.com (A.W.); runzhang822@gmail.com (R.Z.); wanglinjie@sicau.edu.cn (L.W.)

**Keywords:** vascular endothelial growth factor, liver, hepatocyte, AMPK, *Schizothorax prenanti*

## Abstract

Vascular endothelial growth factor A (VEGFA) plays important roles in angiogenesis, inflammatory response as well as energy metabolism in mammals. However, its effect on glycolipid metabolism in fish has not been reported. In this study, we cloned and characterized the *vegfa* gene of *Schizothorax prenanti (S. prenanti)*. *vegfa* expression was significantly higher in liver and muscle than that in other tissues. Then, the VEGFA recombinant protein was expressed in *Escherichia coli* and obtained after purification. VEGFA i.p. injection significantly increased the serum glucose and TG content compared with the control group. Moreover, VEGFA protein aggravated the glycogen and lipid deposition in the liver of *S. prenanti*. In addition, we found that VEGFA treatment increased hepatocyte glycogen and lipid droplet content and increased the levels of pAMPKα (T172). Furthermore, AMPKα inhibition attenuated the ability of VEGFA to induce TG and glycogen accumulation. These results demonstrate that VEGFA regulates hepatic lipid and glycogen metabolism through AMPKα in *S. prenanti*, which may contribute to a better understanding of VEGFA functions in the glycolipid metabolism of fish.

## 1. Introduction

The liver is the main place for carbohydrate and lipid metabolism in fish. Fish livers play a crucial role in the uptake, transportation, metabolism, and excretion of lipids from hepatocytes, as well as acting as storage sites for lipids [1,2]. An imbalance in hepatic lipid homeostasis can lead to excessive hepatic triglyceride accumulation, which causes fatty liver degeneration [3]. Previous studies have proven that high fat feeding induces hepatic lipid deposition, inhibits hepatic antioxidant capacity, and promotes proinflammatory factor expression in fish [4,5]. In addition, fish have poor absorption capacity for dietary carbohydrates. They are susceptible to prolonged hyperglycemia, excessive hepatic lipid accumulation, growth retardation, and decreased immune function after long-term high-carbohydrate diet feeding [6,7,8]. Researchers used [U-14C] glucose as a tracer to prove that ingested glucose is synthesized into glycogen stored in fish livers [9]. Glucose is transported into hepatocytes and metabolized by several pathways, including glycolysis and pentose phosphate synthesis [10].

Vascular endothelial growth factor A (VEGFA) is a member of the vascular endothelial growth factor family, which promotes angiogenesis and induces the proliferation and germination of endothelial cells (ECs) [11]. In zebrafish, VEGFA knockout causes vascular defects in islets [12]. VEGFA has been identified as a biomarker for a number of metabolic diseases due to its involvement in insulin secretion and the regulation of metabolic responses in type 2 diabetes (T2DM) [13,14,15]. In addition, *vegfa* is highly expressed in adipose tissue and is closely related to adipogenesis, adipose tissue development and energy metabolism [16,17]. In mice, VEGFA overexpression in adipose tissue ameliorates diet-induced obesity and insulin resistance [18,19]. The deficiency of VEGFA results in impaired insulin secretion and glucose intolerance [13,20]. The continuous development of aquaculture depends on understanding the energy metabolism of cultured fish [21]. The current intensive farming model with a high-fat and carbohydrate diet will damage the energy homeostasis in the liver of fish [22]. It leads to a disorder of lipid metabolism in fish, which results in fatty liver injury and affects the health of the fish [23]. The role of VEGFA in metabolism is mainly focused on angiogenesis and insulin secretion, whereas little is known about glycolipid metabolism in the liver of fish. Therefore, understanding the molecular mechanism of lipid accumulation in the liver of fish is important for liver health improvement of S. prenanti in artificial cultivation in the future.

*Schizothorax prenanti* (*S. prenanti*) is a Cyprinidae species commonly referred to as the ya-fish. *S. prenanti* is an economically valuable endemic fish species in China, mainly living at the interface between rapid and subcritical streams [24]. It is the cultural symbol of Ya’an city and an important cold-water fish in southwest China [25]. Although VEGFA has been extensively studied for vascular development in fish, the regulation and mechanisms of VEGFA on hepatic glucose and lipid metabolism are still unclear. In the present study, we cloned the *vegfa* gene and obtained VEGFA recombinant proteins of *S. prenanti*. In addition, the effects of VEGFA recombinant proteins on glycolipid metabolism in the liver and hepatocytes were investigated. These results may contribute to a better understanding of VEGFA functions in the glycolipid metabolism of fish.

## 2. Results

### 2.1. Molecular Characterization and Tissue Distribution of VEGFA Gene in S. prenanti

**Figure 1:**
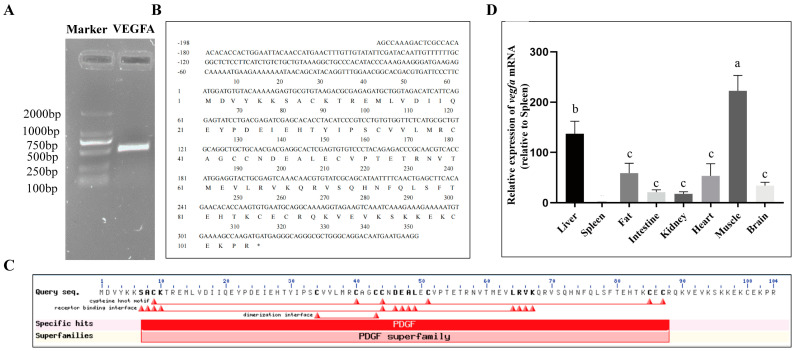
Molecular characterization of *S. prenanti vegfa* gene. (**A**) Gel electrophoresis image of RT−PCR amplification product; (**B**) *vegfa* nucleotide sequences and predicted amino acid sequences; (**C**) the conserved domain of *vegfa* gene of *S. prenanti;* (**D**) tissue distribution of *vegfa* in *S. prenanti*. The data are shown as means ± SEM from *S. prenanti* (*n* = 6). Groups denoted by different letters represent a significant difference (*p* < 0.05).

**Figure 2:**
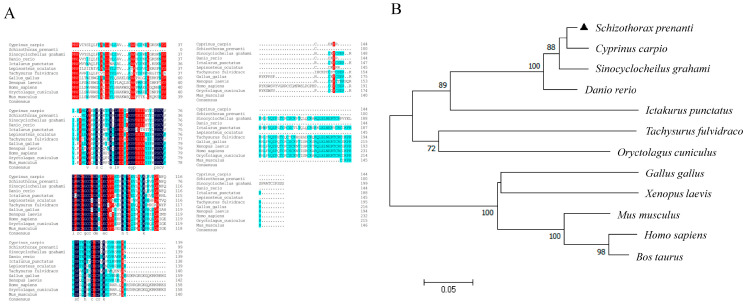
Homology analysis of *S. prenanti* VEGFA. (**A**) Homology alignment of the amino acid sequence of VEGFA from different species. Black area: 100%, blue area: ≥75%, red area: ≥50% homology. (**B**) Phylogenetic tree comparing VEGFA of *S. prenanti* with other vertebrates using MEGA6. Scale bar represents genetic distance as a percent difference (0.05 = 5%). The numbers on the nodes are bootstrap values, which indicate branch support on the phylogenetic tree.

The length of the *S. prenanti vegfa* gene is 549 bp; it encodes a predicted protein of 104 amino acids with a calculated molecular mass of 25.23 kDa and theoretical isoelectric point (pI) of 8.91 (Figure 1A,B). The VEGFA protein contains a conserved domain, belonging to the PDGF superfamily (Figure 1C). In addition, we found that *vegfa* expression was higher in the muscle and liver than that in other tissues (*p <* 0.05) (Figure 1D). Alignment of the VEGFA amino acid sequences showed that *S. prenanti* VEGFA exhibited high homology with *Cyprinus carpio* (95.8%), *Sinocyclocheilus graham* (92.36%) and *Danio rerio* (93.06%). The phylogenetic trees showed that the VEGFA protein of *S. prenanti* was close to several other teleost relatives, with distinct branches from other mammals, amphibians, and birds, largely consistent with the animal classification during biological evolution (Figure 2A,B).

### 2.2. Production of Recombinant S. prenanti VEGFA Protein

**Figure 3:**
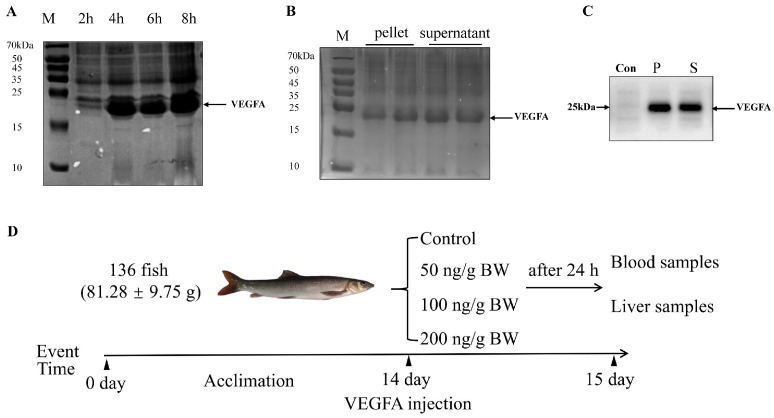
The expression and purification of recombinant VEGFA protein of *S. prenanti*. (**A**) The expression of recombinant VEGFA protein induced for different time. M: protein marker (**B**) SDS-PAGE of the purified protein of VEGFA. Lane M: protein marker. Recombinant VEGFA proteins were expressed in both the pellet (P) and supernatant (S) fractions. (**C**) The expressed proteins were verified by western blotting with anti-6 × His-tagged antibody in Pellet (P) and supernatant (S) fractions. Bacteria lysates from absence of IPTG induction were used as negative control (Con). (**D**) Flow chart showing the experimental design timeline.

To obtain the VEGFA recombinant protein, specific expression bacteria were stimulated by IPTG at 37 °C for 2, 4, 6, 8 h, respectively. The molecular mass of the recombinant VEGFA protein was approximately 25 kDa with an N-terminal polyhistidine (6 × His) tag. The expression of the VEGFA protein was higher at 8 h of 1 mM IPTG treatment than other treatments (Figure 3A). Furthermore, we found that recombinant VEGFA proteins were expressed in both the pellet and supernatant fractions using SDS-PAGE analysis (Figure 3B). Then, the recombinant VEGFA proteins were obtained after purification by Ni^+^-NTA columns. Finally, the VEGFA protein could be detected by 6 × His-tag antibody (Figure 3C), indicating that it is the 6 × His-tag recombinant fusion protein.

### 2.3. Biochemical Analysis of Plasma in S. prenanti after Intraperitoneal Injection of VEGFA Protein

In this study, a total of 136 fish were randomly divided into four groups, and each group was i.p. injected with different concentrations of recombinant VEGFA protein, respectively (Figure 3D). To determine the effect of VEGFA on glycolipid metabolism in *S. prenanti*, we performed biochemical assays on *S. prenanti* serum after intraperitoneal injection of recombinant protein VEGFA at different concentrations. As shown in Table 1, the blood glucose content was significantly upregulated at the protein concentration of 100 ng/g BW after intraperitoneal injection of 24 h (*p* < 0.05). Meanwhile, 100 and 200 ng/g BW VEGFA i.p. injection significantly increased the total cholesterol (TC) and triglycerides (TG) content compared with control group (*p* < 0.05). 

### 2.4. Effects of VEGFA Protein on Hepatic Lipid and Glycogen Metabolism in S. prenanti

**Figure 4:**
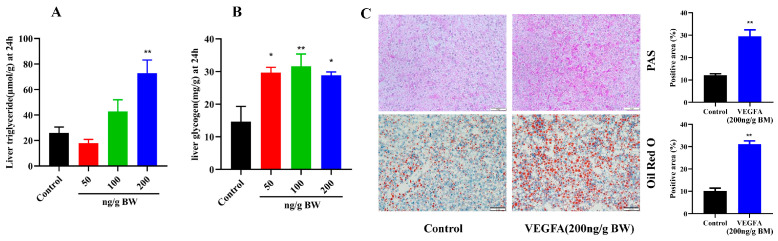
Effect of VEGFA protein on hepatic triglycerides and glycogen of *S. prenanti*. Fish were injected intraperitoneally with vehicle (PET-32a, control) or recombinant VEGFA (50, 100, and 200 ng/g BW) for 24 h after i.p. injection. Data are shown as means ± SEM, n = 6, * *p* < 0.05, ** *p* < 0.01. (**A**) Changes in *S. prenanti* liver triglyceride (n = 6). (**B**) Changes in *S. prenanti* liver glycogen. (**C**) PAS staining and Oil red staining of liver after VEGFA protein i.p. injection. Scale bar: 50 μm.

To further investigate the roles of VEGFA on hepatic glycolipid metabolism in *S. prenanti*, we determine hepatic glycogen and TG content. As shown in the Figure 4A, there was a significantly higher triglyceride content compared with the control group at 24 h after 200 ng/g BW VEGFA treatment (*p* < 0.01). There was no difference in the levels of TG content at a relatively low dose (50 ng/g BW and 100 ng/g BW) compared with the control group (Figure 4A). Meanwhile, all three doses of VEGFA significantly (*p* < 0.05) elevated the glycogen content of liver compared with the control group (Figure 4B). Next, we found that the glycogen and lipid accumulation in liver was increased after 200 ng/g BW VEGFA i.p. injection by PAS staining and Oil red O staining (Figure 4C). 

**Figure 5:**
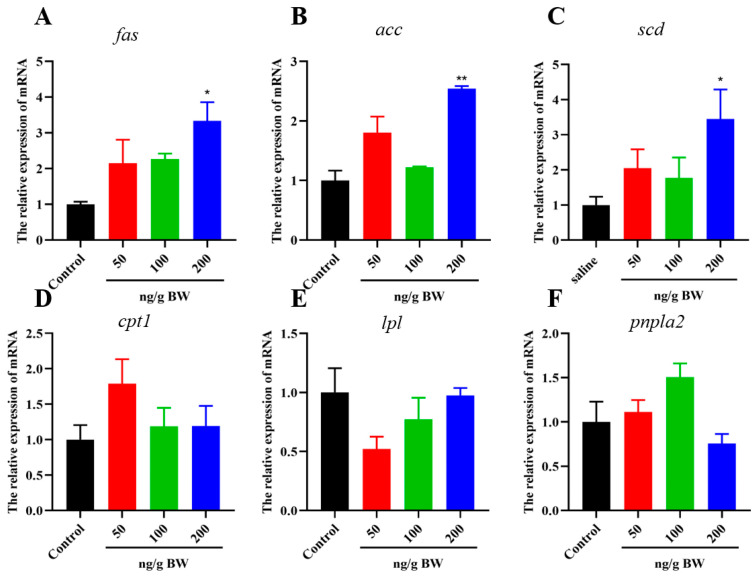
Effects of VEGFA protein on the expression of genes related to lipid metabolism in liver. After i.p. injection of recombinant VEGFA protein (50, 100, and 200 ng/g BW), samples were collected and the genes expression were detected. Changes in mRNA expression of lipid metabolism-related genes after i.p. (**A**) *fas*, (**B**) *acc*, (**C**) *scd1*, (**D**) *cpt1*, (**E**) *lpl*, and (**F**) *atgl*. Data are shown as means ± SEM, n = 6, * *p* < 0.05, ** *p* < 0.01.

Then, expression levels of genes related to glycolipid metabolism were measured with qPCR. We found that the expression levels of genes encoding key enzymes of lipogenesis (*fas*, *acc* and *scd1*) were increased (*p* < 0.05) at high dose of 200 ng/g BW compared with the control group (Figure 5A–C). There was no significant effect on the expression of lipolysis-related genes (*cpt1*, *lpl*, and *atgl*) at three doses of the VEGFA protein (Figure 5D–F).

**Figure 6:**
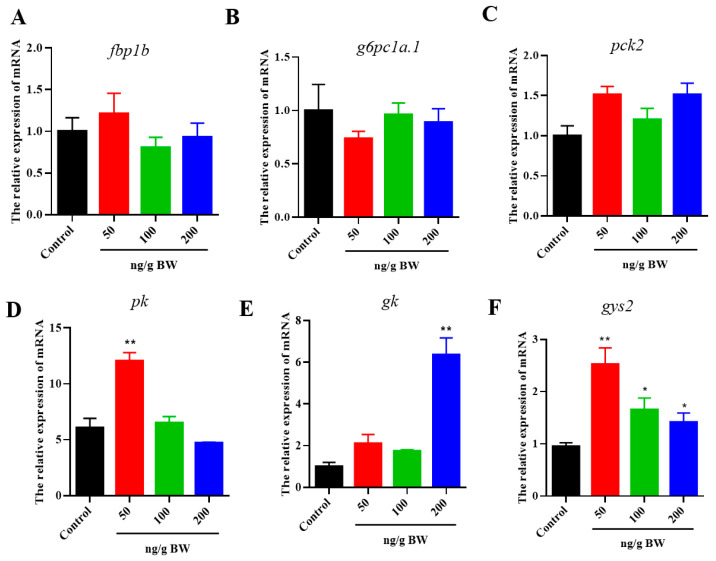
Effects of VEGFA proteins on the expression of genes related to glycogen metabolism. Changes in mRNA expression of glycogen metabolism-related genes. (**A**) *FBPase*, (**B**) *G6Pase*, (**C**) *pepck*, (**D**) *pk*, (**E**) *gk*, and (**F**) *gys2*. Data are shown as means ± SEM. n = 6, * *p* < 0.05, ** *p* < 0.01.

For gluconeogenesis, three doses of VEGFA protein did not affect the expression levels of genes encoding key enzymes of gluconeogenesis (*FBPase*, *G6Pase*, and *pepck*) (Figure 6A–C). For glycolysis, *pk* and *gk* expression were significantly (*p* < 0.05) up-regulated at doses of 50 and 200 ng/g BW group compared with the control group, respectively (Figure 6D,E). Moreover, liver glycogen synthase gene (GYS2) expression was significantly (*p* < 0.05) upregulated at three doses of VEGFA i.p. injection (Figure 6F).

### 2.5. Effects of VEGFA Protein on Lipid and Glycogen Metabolism in Hepatocytes

**Figure 7:**
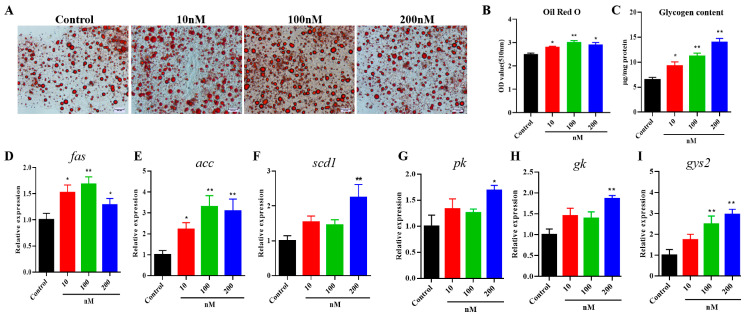
Effect of VEGFA proteins on lipid and glycogen content of hepatocytes. (**A**) The Oil red O staining of hepatocytes after VEGFA protein treatment (scale bar of 100 μm). (**B**) Lipid droplets were quantified by absorbance at 510 nm after extraction with isopropanol. (**C**) Changes in glycogen of hepatocytes after VEGFA treatment. Expression levels of lipid and glycogen metabolism-related genes. (**D**) *fas*, (**E**) *acc*, (**F**) *scd1*, (**G**) *pk*, (**H**) *gk*, and (**I**) *gys2*. Data are shown as means ± SEM. n = 6, * *p* < 0.05, ** *p* < 0.01.

We further investigate the roles of VEGFA on triacylglycerol and glycogen deposition in primary hepatocytes of *S. prenanti*. The TG and glycogen content measurement was performed on hepatocytes. As shown in Figure 7A–C, VEGFA remarkedly increased lipid and glycogen accumulation in hepatocytes in a dose-dependent manner with the effective dose for VEGFA effects from 10 to 200 μΜ (*p* < 0.05). In addition, compared with the control group, VEGFA increased the expression levels of *fas* and *acc* genes at three doses from 10 to 200 μΜ (*p* < 0.05) (Figure 7D,E), whereas *scd1* was upregulated only by 200 nM VEGFA treatment (Figure 7F). *pk*, *gk*, and *gys2* genes expression were significantly (*p* < 0.05) increased at doses of the 100 and 200 μΜ groups compared with the control group (Figure 7G–I).

### 2.6. VEGFA Regulates Glycolipid Metabolism through AMPKα Signaling in Hepatocytes

**Figure 8:**
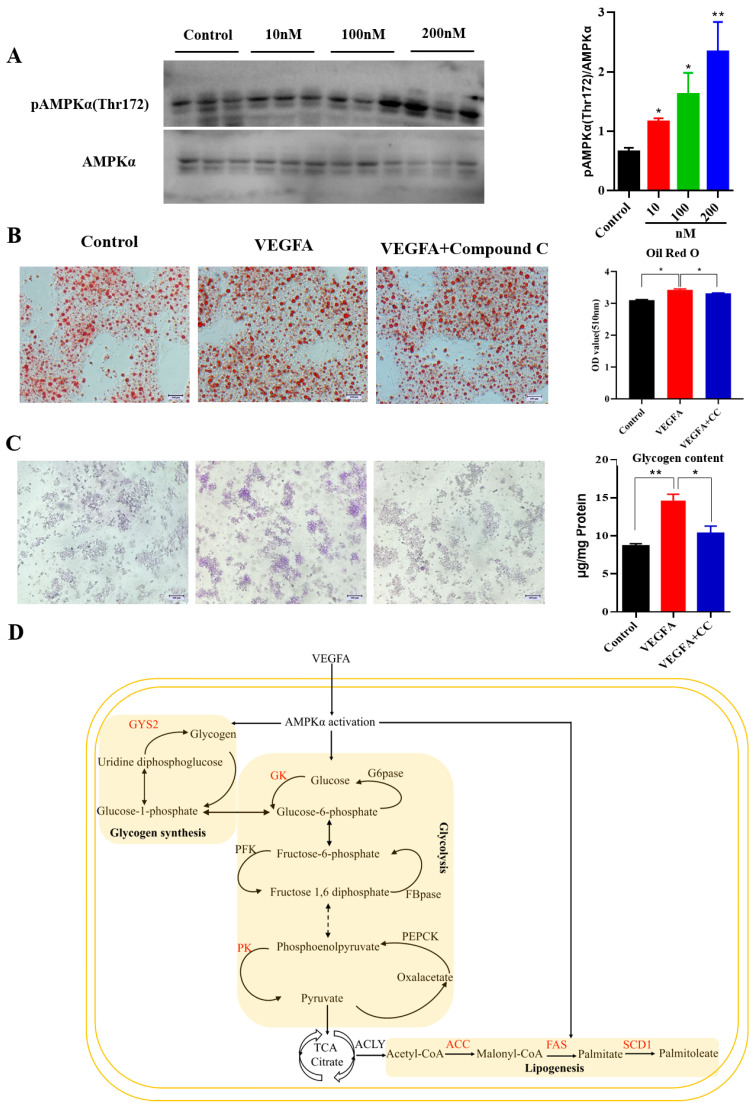
VEGFA regulates glycolipid metabolism through AMPKα signaling in hepatocytes. (**A**) Western blot and quantitative analysis of pAMPKα (T172). Different concentrations of recombinant VEGFA proteins (0, 10, 100, and 200 nM) were added in hepatocytes (n = 3). (**B**) Oil red O staining and quantification of hepatocytes under VEGFA protein and compound C treatment (n = 6). (**C**) PAS staining and glycogen quantification of hepatocytes treated with VEGFA and compound C (n = 6). Scale bar: 50 μm. (**D**) The schematic diagram of VEGFA activating AMPKα signaling pathway in liver. Red represents up-regulated genes.

To determine whether VEGFA regulated AMPKα activation, different concentrations of VEGFA recombinant proteins (0, 10, 100, and 200 nM) were added in hepatocytes. As shown in Figure 8A, VEGFA treatment significantly induced the levels of pAMPKα (T172), reaching a peak at 200 nM (*p* < 0.01). Furthermore, 200 nM VEGFA treatment increased hepatocyte glycogen and lipid droplet content (*p* < 0.01). Then, we examined whether AMPKα inactivation by compound C could rescue the TG and glycogen accumulation. We found that AMPKα inhibition attenuated the ability of VEGFA to induce TG and glycogen accumulation (*p* < 0.01) (Figure 8B,C). These results indicate that VEGFA regulates glycogen and lipid deposition through AMPKα in hepatocytes (Figure 8D).

## 3. Discussion

It is well known that VEGFA plays important roles in angiogenesis, mitosis, and enhanced vascular permeability [11,26]. Recently, the studies of VEGFA have been extended from mammals to fish models. Previous studies have cloned the *vegfa* gene in the hindgut of loach (*Misgurnus anguillicaudatus*) and zebrafish [27,28,29]. Additionally, it has been demonstrated that the coding region of the *vegfa* gene is highly conserved from teleost fish (zebrafish and pufferfish) to mammals [30]. In this study, we found that the amino acid sequence of VEGFA of *S. prenanti* had low homology to mammals but was highly conservative with other fish based on amino acid sequence alignment. In zebrafish, VEGFA regulates endothelial cell permeability and vasculogenesis as an angiogenic growth factor [31]. Meanwhile, studies have proven that VEGFA is closely related to the normal function of blood vessels in various tissues, such as the brain, liver, muscle, and heart [32,33,34]. Therefore, *vegfa* is widely distributed in various tissues of animals such as the brain, heart, liver as well as muscle [34,35,36]. In this study, we found that *vegfa* expression was higher in the muscle and liver than that in other tissues, indicating that VEGFA may play an important role in muscle and liver metabolism of *S. prenanti*. 

A previous study has found that VEGFA plays important roles in adipose tissue energy metabolism and promotes adipose tissue lipolysis [37]. VEGFA up-regulates the expression of *PGC1α* and *UCP1* genes, which causes browning of white adipose tissue and an increase in energy expenditure [38,39]. VEGFA transgenic mice show higher thermogenesis and energy expenditure in brown adipose tissue (BAT) after cold exposure compared with wild type (WT) mice [38,40]. Recent studies have also demonstrated the different roles of VEGFA in hepatic lipid metabolism. VEGFA inhibition reduces in triglyceride content in the liver and muscle induced by the high-fat feeding [41], which suggests that the VEGFA protein may promote lipid accumulation in liver and muscle. In the *S. prenanti*, we showed that VEGFA protein increased serum glucose and TG content, and aggravated lipid deposition by up-regulating the expression levels of lipogenesis-related genes (*fas*, *acc*, and *scd1*). Furthermore, we found that VEGFA protein had no effects on the expression of lipolysis-related genes (*lpl* and *atgl*), indicating that VEGFA induces lipid accumulation in liver of *S. prenanti* by promoting the lipogenesis process. 

Recent studies have demonstrated that VEGFA transgenic mice show reduced blood glucose content, improved insulin sensitivity, and glucose tolerance in high-fat diet (HFD)-challenged mice [38,42]. In addition, BAT-specific VEGFA transgenic mice display improved glucose and lipid metabolism as well as a decrease in HFD-induced hepatic steatosis [40]. Previous studies have shown that VEGFA can further reduce cell viability and increase cell damage to enhance high glucose induced glucotoxicity in mammals [43,44], indicating that VEGFA may promote glycogen synthesis during glucose metabolism. In the present study, we found that 200 ng/g BW VEGFA i.p. injection significantly increased the serum glucose content compared with the control group. VEGFA significantly elevated the glycogen content of liver compared with the control group. In addition, VEGFA recombinant protein up-regulated *gys2*, *gk*, and *pk* of glycolysis-related key genes. The above results suggest that VEGFA may regulate hepatic glycogen content of *S. prenanti* by regulating glycolysis and the glycogen synthesis process. 

AMPK is an important protein kinase in the regulation of energy metabolism, which increases ATP production by promoting glycolipid catabolic processes and preserves ATP by mechanistic glycolipid biosynthetic pathways [45]. Previous studies have shown that AMPK is the energy status sensor in regulating the glucose and lipid metabolism. In mice, AMPK activation increased glucose uptake and glycogen storage while contraction-induced glucose uptake leads to increased lactate levels and glucose oxidation [46]. The knockout of AMPK increases energy expenditure and protects against diet-induced obesity, glucose intolerance and insulin resistance [47]. AMPK promotes fatty acid oxidation and suppresses lipid synthesis in muscle satellite cells by phosphorylating several substrates, including ACC, ATGL, and HSL [48,49]. In the present study, we proved that VEGFA treatment increased the levels of pAMPKα (T172). 

VEGFA activated the hepatic AMPKα signaling pathway to upregulate the expression of glycogen synthesis gene (*gys2*) and lipogenesis genes (*acc*, *fas* and *scd1*), promoting glycogen synthesis and lipid accumulation in the liver. The accumulation of hepatic lipids increased after VEGFA injection, which may be the reason for the increase in triglycerides and total cholesterol levels in the blood. We further found that AMPKα inhibition attenuated the ability of VEGFA to induce TG and glycogen accumulation in hepatocytes. These results suggested that VEGFA regulates glycolipid metabolism through the AMPKα signaling pathway. 

*S. prenanti* is a valuable cold-water fish species in aquaculture in western China. Fish typically have a limited ability to digest, absorb, and metabolize carbohydrates and fat. Therefore, feeding excess carbohydrate and fat diets may result in a metabolic burden on fish due to increased hepatic glycogen and lipid deposition and prolonged hyperglycemia [50], which may suppress immune function and increase susceptibility to infectious diseases [51]. Modern aquaculture needs to understand the glucose and lipid metabolisms that determine the growth and health status of cultured fish. According to our studies, VEGFA regulates hepatic glucose and lipid metabolism in hepatocytes through the AMPKα signaling pathway. This study provides a reference for further understanding the effects of VEGFA on glucose and lipid metabolism in the liver of *S. prenanti*.

## 4. Materials and Methods

### 4.1. Animals and Intraperitoneal Injection of VEGFA Recombinant Protein

All research involving animals was conducted by the Institutional Animal Care and Use Committee at Sichuan Agricultural University, under permit No. DKY-B2020302177. The *S. prenanti* were cultivated at the Fish Breeding Center of Sichuan Agriculture University (Ya’an, China) and kept at 17 ± 1 °C. Fish were acclimated to laboratory conditions for two  weeks before being used in experiments. Fish were fed 1.0% body weight twice daily (9:00 a.m. and 6:00 p.m.) with commercial food (G68, Sichuan, China) and maintained a constant photoperiod of 12 h (8 a.m.–8 p.m.). To determine tissue expression patterns, eight tissues (liver, brain, adipose, intestine, spleen, kidney, heart and muscle) were collected from *S. prenanti* (one biological replicate of n = 6) after anesthesia with MS222 (80 mg/L).

To determine the roles of VEGFA protein on glycogen and lipid metabolism of *S. prenanti*, intraperitoneal (i.p.) injections of VEGFA recombinant protein were preceded. A total of 136 fish (81.28 ± 9.75 g) were randomly divided into four groups (n = 34, each group). Each group was i.p. injected with 0 and 50, 100, 200 ng/g BW of recombinant VEGFA protein, respectively. Fish were sacrificed with MS222 anesthesia (80 mg/L) after 24 h of injection. Blood samples were collected from the tail vein and centrifuged at 1000× *g* for 15 min at 4 °C to obtain serum samples, which were stored at −80 °C until analyses. Eight tissues of liver, brain, adipose, intestine, spleen, kidney, heart and muscle were collected and stored at −80 °C. 

### 4.2. Blood Biochemical Assays

Serum biochemical parameters including total cholesterol (TC), triglycerides (TG) and glucose were measured using a Hitachi 7020 automatic biochemical analyzer (Hitachi, Tokyo, Japan).

### 4.3. Oil Red O and PAS Staining

Liver tissues were fixed in 4% buffered paraformaldehyde (pH 7.4). Then, sections were stained with Oil Red O solution [52] (Sigma, MA, USA) and Periodic Acid-Schiff solution [53] (Solarbio, Beijing, China). The slides were washed twice with 1 × PBS for 5 min and air-dried before being sealed with glycerol gelatin (Sigma-Aldrich, Saint-Louis, MO, USA). Finally, sections were photographed using a microscope (Nikon, Tokyo, Japan) with NIS Elements 4.13 software (Nikon). ImageJ version 1.48 software (NIH) was used to quantify the Oil red O and PAS-positive staining.

### 4.4. Triglyceride and Glycogen Content Measurement

Liver triglyceride and glycogen content were measured using the Triglyceride and Glycogen Assay Kit [53] (Jiancheng Biotech Co, Nanjing, China). Briefly, liver samples were treated with anhydrous ethanol (Analytical Pure, Kelong Chemical, Chengdu, China) and incubated with glycerol-3-phosphate oxidase-phenol + aminophenazone (GPO-PAP) for 10 min at 37 °C, and the absorbance value was determined at 510 nm. The chromogenic solution was mixed with the glycogen detection solution then incubated at 100 °C for 5 min, and the absorbance was measured at 620 nm.

### 4.5. Cloning of S. prenanti VEGFA and Sequence Analysis

Six liver tissues were cut into pieces and snap-frozen for homogenization to extract total liver RNA using the Total RNA Extraction Kit for Animal Tissues (Foregene, Chengdu, China). The concentration and purity of RNA were determined using a NanoDrop-1000 Spectrophotometer (Thermo Scientific, Wilmington, DE, USA). A single total RNA ≥ 1 µg, concentration ≥ 35 ng/µL, OD 260/280 ≥ 1.8, and OD 260/230 ≥ 1.0. Then, cDNA was synthesized from 1 µg of total RNA by using the PrimeScript™ RT Reagent kit (Takara, Dalian, China). Primer Premier 6.0 was used to design primers to amplify cDNA sequences based on our full-length transcripts of *S. prenanti* [54]. The PCR products were purified using a PCR purification kit (TianGen kit, China) and ligated to the pMD19-T vector. Then, construct was transformed into the *E. coli* DH5α and the sequence was verified by Sanger sequencing (Sangon Biotech, Shanghai, China). Open reading frames (ORFs) were predicted using the ORF Finder tool (https://www.ncbi.nlm.nih.gov/orfnder/ (accessed on 25 July 2023)). The conserved domains were predicted using the NCBI Conserved Domain Database (https://www.ncbi.nlm.nih.gov/Structure/cdd/wrpsb.cgi/ (accessed on 28 July 2023)). Multiple sequences were aligned and phylogenetic analysis were performed using MEGA 6.0 software.

### 4.6. Expression of VEGFA Recombinant Protein and Purification

PCR amplification was performed using double restriction sites (*Bam*HI and *Hind*III) with the program of denaturation at 95 °C for 2 min followed by 40 cycles of 95 °C for 5 s, 54.2 °C for 30 s, 10 s at 72 °C, and a final extension at 72 °C for 5 min. The PCR products were purified and subcloned into pET32a (+) His-tag expression vector. Then pET32a-VEGFA was transformed into *E. coli* BL21 (DE3) competent cells and the sequence was verified by Sanger sequencing. To determine the appropriate conditions for the induction of recombinant VEGFA protein, 1 mM isopropyl-β-d-thiogalactopyranoside (IPTG) [55,56] (Solarbio, China) were added and incubated (37 °C) at 2, 4, 6, and 8 h, respectively. Following incubation with appropriate duration, the bacteria were centrifuged at 3000 rpm for 15 min at room temperature and washed once with 1 × PBS (pH 8.0).

To determine the solubility of VEGFA protein, the bacteria were resuspended in PBS (pH 8.0) and was ultrasonically crushed in an ice bath. The cell lysates were separated by centrifugation at 12,000 rpm for 20 min at 4 °C. The precipitate and supernatant were assessed by 10% SDS-PAGE and stained by the Coomassie brilliant blue (Solarbio, Beijing, China). The supernatant was purified by Capturem^TM^ His-tagged purification kit (Takara, Dalian, China). The recombinant protein produced was tested by SDS-PAGE and quantified using BCA protein assay kit (BestBio, Shanghai, China). 

### 4.7. Primary Hepatocytes Culture and Recombinant VEGFA Protein Treatment

After body surface wiping with a 75% alcohol cotton ball, liver tissues were sampled under aseptic conditions and digested in 0.25% Trypsin-EDTA (Gibco, CA, USA) for 5 min at 25 °C. The digestion mixture was filtered with 70 μm and 40 μm nylon filters, respectively. After centrifugation at 1000 rpm/min (4 °C) for 5 min, the harvested cell pellets were suspended in PBS. Finally, cells were placed into six-well no-coated plates (Corning, NY, USA) with M199 medium (Gibco, CA, USA) with 15% fetal bovine serum, and cultured at 25 °C in a humidified incubator containing 5% CO_2_. After the hepatocytes were grown to 80% confluence, the cells were incubated with different doses of VEGFA recombinant protein (0, 10, 100, and 200 nM) for 24 h. To investigate whether VEGFA recombinant protein regulates glycolipid metabolism in hepatocytes through AMPK pathway, hepatocytes were incubated with 100 nM VEGFA recombinant protein and 10 μM compound C (AMPK inhibitor) (ABclonal, Wuhan, China) for 24 h. 

### 4.8. qPCR

The cDNA was synthesized by the HiScript^®^ III RT SuperMix for qPCR (Vazyme, Nanjing, China). qPCR was conducted in a final volume of 10 μL (5 μL of SYBR qPCR Master, 3.4 μL of sterilized double-distilled water, 0.8 μL of cDNA and 0.4 μL (0.4 mM) of each primer) using a CFX96TM Real-Time PCR Detection System (Bio-Rad, Hercules, CA, USA). The cycling conditions were 60 s at 95 °C, followed by 40 cycles of 95 °C for 10 s, 60 °C for 30 s. Hepatic glycolipid metabolism-related genes expression was normalized by the 2^−ΔΔCt^ method [57]. The reference genes *β-actin* and *GAPDH* were considered as house-keeping genes [58,59]. All experiments were performed with six replicates. Primers (Table S1) were designed using Primer-Blast, and specificity was confirmed using BLAST (NCBI). In addition, melting curve analysis was used to confirm specific PCR products.

### 4.9. Western Blotting

The protein of liver was extracted by the total protein extraction kit (Solarbio, Beijing, China). The protein concentration of the lysate was determined by the BCA protein detection kit (BestBio, Shanghai, China). Antibodies were diluted at 1:1000 for mouse anti-Phospho-AMPKα (Thr172) and mouse anti-AMPKα (Cell Signaling Technology, Beverly, MA, USA), 1:1000 for rabbit anti-β-Tubulin and HPR-labeled goat anti-mouse IgG (Abclonal, Wuhan, China). 

### 4.10. Statistical Analysis

All data are expressed as mean ± SEM and were analyzed by paired or unpaired Student’s *t*-tests using SPSS 19.0 software (IBM Corporation, Armonk, NY, USA). *p* < 0.05 was considered statistically significant, and *p* < 0.01 was considered highly statistically significant.

## 5. Conclusions

In summary, we isolated and purified the VEGFA protein of *S. prenanti*. In vivo and in vitro studies have shown that VEGFA increased the content of TG and glycogen. Additionally, we have shown that VEGFA regulates the glycolipid metabolism through the AMPKα signaling pathway. These findings show that VEGFA plays an important role in the regulation of the hepatic glycolipid metabolism in *S. prenanti*, which provides the basis for a comprehensive understanding of VEGFA functions in the glycolipid metabolism in teleosts.

## Figures and Tables

**Table 1:** Plasma parameters of *S. prenanti* after intraperitoneal injection of VEGFA protein.

Plasma Parameters	Control	50 ng/g BW	100 ng/g BW	200 ng/g BW
Glucose (mmol/L)	7.54 ± 0.74	7.32 ± 0.83	15.46 ± 2.84 *	6.75 ± 0.59
TG (mmol/L)	2.94 ± 0.14	3.32 ± 0.07	3.67 ± 0.16 *	4.03 ± 0.27 *
TC (mmol/L)	7.02 ± 0.94	8.04 ± 1.27	8.84 ± 1.16 *	9.45 ± 1.07 *

Values are mean ± SEM, n = 6. * *p* < 0.05 relative to control. Total cholesterol (TC) and triglycerides (TG).

## Data Availability

Not applicable.

## Supplementary Materials

The following supporting information can be downloaded at: https://www.mdpi.com/article/10.3390/ijms242015171/s1.
