# Supplementary material for: Vascular Endothelial Growth Factor A (VEGFA) Regulates Hepatic Lipid and Glycogen Metabolism in Schizothorax prenanti
Source: Int J Mol Sci. 2023 Oct 14;24(20):15171. doi: 10.3390/ijms242015171 (PMC10606705; doi:10.3390/ijms242015171)
Supplement: Supplementary file 1 [file ijms-24-15171-s001.zip › ijms-2602576-supplementary.pdf]

Table S1 Sequences of primers used in this study

Primer names	Primer sequences (5'-3')	Tm (℃)
VEGFA-F	AGCCAAAGACTCGCCACAAC	54.2
VEGFA-R	TGGGCAGGACAATGAATGAAGG	
VEGFA-F（BamHI）	CGCGGATCCATGTCGTCAGATGTCACCAA	57
VEGFA-R（HindIII）	CCCAAGCTTGTCTTTGTGCAAAGTCT	
GK-F	AGAGGAAGTGATGAGGAGAA	57
GK-R	GTGGAGCGGACATAAGTG	
PK-F	GCACTCATACAACAGCCTTAGC	57
PK-R	TCCTGGAGCATCTGTGTCTG	
G6Pase-F	CCTGAAGGCTCTCGGTGTGGAT	57
G6Pase-R	ACTTGCGAACGGTGTGGTGTC	
FBPase-F	AGATGGCAGTTCACCTTATGGA	57
FBPase-F	GCACTCATACAACAGCCTTAGC	
PEPCK-F	TGTCCTTCGGCAGCGGTTAC	55.7
PEPCK-R	CAGCCAACCAGCCTTCGTCTT	
GYS2-F	CAGAGGAACCGAACAGAGCG	59.9
GYS2-R	TGAGGAGATGAGTGAAGCGAGTG	
FAS-F	CAGTAGTGTATGCCACCGCA	59
FAS-R	TGCAATAGCAATAGCGGCCT	
ACC-F	GGGCACAAAGACCGACAGAT	55
ACC-R	GCCTGGCGAAACATTTCTGG	
SCD1-F	TGTGCTGCTGATGTGCTT	59.4
SCD1-R	GATGTTGCCGTCATAGGG	
LPL-F	ATGGACGGTCACGGGTAT	59
LPL-R	TTTGGTGTAGGCGGCAGA	
CPTIA-F	TGAGTGATTGGTGGGAGG	59
CPTIA-R	GCATGGCATGGACTACGT	
ATGL-F	CTGGGTATTTATCACATCGG	59
ATGL-R	GCTTCTTTAGCCACGTCAA	
GAPDH-F	TGACCCGTGCTGCTTTC	55
GAPDH-R	GCCTTAACCTCACCCTTGT	
β-actin-F	TTCTTGGGTATGGAGTCTTG	59
β-actin-R	AGGTCCTTACGGATGTCG	
